# Normal values of aortic dimensions, distensibility, and pulse wave velocity in children and young adults: a cross-sectional study

**DOI:** 10.1186/1532-429X-14-77

**Published:** 2012-11-14

**Authors:** Inga Voges, Michael Jerosch-Herold, Jürgen Hedderich, Eileen Pardun, Christopher Hart, Dominik Daniel Gabbert, Jan Hinnerk Hansen, Colin Petko, Hans-Heiner Kramer, Carsten Rickers

**Affiliations:** 1 Department of Congenital Heart Disease and Pediatric Cardiology, University Hospital of Schleswig-Holstein, Campus Kiel, Arnold-Heller-Str. 3, 24105, Kiel, Germany; 2 Department for Medical Informatics and Statistics, University Hospital of Schleswig-Holstein, Campus Kiel, Arnold-Heller-Str. 3, 24105, Kiel, Germany; 3 Department of Radiology, Brigham & Women's Hospital, Harvard University, 75 Francis Street, Boston, MA, 02115, USA

## Abstract

**Background:**

Aortic enlargement and impaired bioelasticity are of interest in several cardiac and non-cardiac diseases as they can lead to cardiovascular complications. Cardiovascular magnetic resonance (CMR) is increasingly accepted as a noninvasive tool in cardiovascular evaluation. Assessment of aortic anatomy and bioelasticity, namely aortic distensibility and pulse wave velocity (PWV), by CMR is accurate and reproducible and could help to identify anatomical and bioelastic abnormalities of the aorta. However, normal CMR values for healthy children and young adults are lacking.

**Methods:**

Seventy-one heart-healthy subjects (age 16.4 ± 7.6 years, range 2.3 - 28.3 years) were examined using a 3.0 Tesla CMR scanner. Aortic cross-sectional areas and aortic distensibility were measured at four positions of the ascending and descending thoracic aorta. PWV was assessed from aortic blood flow velocity measurements in a aortic segment between the ascending aorta and the proximal descending aorta. The Lambda-Mu-Sigma (LMS) method was used to obtain percentile curves for aortic cross-sectional areas, aortic distensibility and PWV according to age.

**Results:**

Aortic areas, PWV and aortic distensibility (aortic cross-sectional areas: r = 0.8 to 0.9, p < 0.001; PWV: r = 0.25 to 0.32, p = 0.047 to 0.009; aortic distensibility r = -0.43 to -0.62, p < 0.001) correlated with height, weight, body surface area, and age. There were no significant sex differences.

**Conclusions:**

This study provides percentile curves for cross-sectional areas, distensibility and pulse wave velocity of the thoracic aorta in children and young adolescents between their 3^rd^ and 29^th^ year of life. These data may serve as a reference for the detection of pathological changes of the aorta in cardiovascular disease.

## Background

The thoracic aorta plays an important role in the cardiovascular system. It’s elastic buffering capacity transforms the pulsatile effect caused by ventricular ejection into a continuous blood flow [1,2]. In children and young adults several cardiac and non-cardiac diseases manifest themselves by aortic enlargement and impaired aortic bioelastic function [3-6]. These changes may be of clinical relevance as they can lead to cardiovascular complications such as left ventricular dysfunction [7], aneurysm formation, atherosclerosis, myocardial infarction and stroke [8-10] in later life.

Aortic distensibility and aortic pulse wave velocity (PWV) are two parameters closely related to the bioelastic function of the aorta and serve as pathogenic markers in cardiovascular disease [11]. Quantification of aortic distensibility and PWV by cardiovascular magnetic resonance (CMR) has been shown to be accurate and reproducible and could help in identifying early cardiovascular disease in asymptomatic patients [1,12,13].

However, reference ranges from childhood to adulthood are lacking. Therefore we sought to establish CMR normal ranges of aortic distensibility and aortic PWV as well as of aortic cross-sectional areas in heart-healthy children and young adults.

## Methods

### Study population

71 children and young adults aged 2.3 - 28.3 years underwent a CMR study for the assessment of aortic dimensions, distensibilty and PWV. Table 1 shows the sex and age distribution of the total study group.

**Table 1:** Sex and age distribution of the study group

			**Age classes (years)**						**Total**
			**-5**	**5 - 10**	**10-15**	**15-20**	**20-25**	**25-30**	
Sex	male	n	2	10	7	2	6	3	30
		%	6.7%	33.3%	23.3%	6.7%	20.0%	10.0%	100.0%
	female	n	2	7	7	6	11	8	41
		%	4.9%	17.1%	17.1%	14.6%	26.8%	19.5%	100.0%
Total study group		n	4	17	14	8	17	11	71
		%	5.6%	23.9%	19.7%	11.3%	23.9%	15.5%	100.0%

The study participants were recruited among medical students and healthy children of hospital staff. Five children were recruited from the department of pediatric neurology. They underwent diagnostic magnetic resonance imaging (MRI) of the central nervous system (CNS) because of psychomotor retardation and epilepsy. Immediately after CNS MRI, non-contrast enhanced CMR was performed. All study subjects were free from cardiovascular disease. During the study, heart rate, respiratory motion, oxygen saturation and non-invasive blood pressure were monitored.

The study protocol was approved by the local research ethics committee and conformed to the principles outlined in the Declaration of Helsinki. Written informed consent was obtained from participants older than 17 years and all persons responsible for care and custody of the child.

### Image acquisition

All CMR studies were performed with a 3.0 Tesla CMR scanner (Achieva 3.0 T, Philips Medical Systems, Netherlands) using a phased-array coil for cardiac imaging (SENSE™ Cardiac coil, Philips Medical Systems, Netherlands).

Gradient echo cine CMR with retrospective gating was applied to assess aortic cross-sectional areas, which were used to describe the normal dimensions of the aorta and for distensibility calculation. We collected axial and coronal stacks of parallel, contiguous, views perpendicular to the aortic axis. The scan parameters were as follows: 280 × 224 mm, voxel size (read-, phase-encoding, and slice directions) 1.88 × 1.94 × 6 mm, TR/TE = 4.4/2.5 ms, 25 cardiac phases, no inter-slice gap, non-breath-hold, number of repetitions: 2, scan duration: 3-6 min.

**Figure 1:**
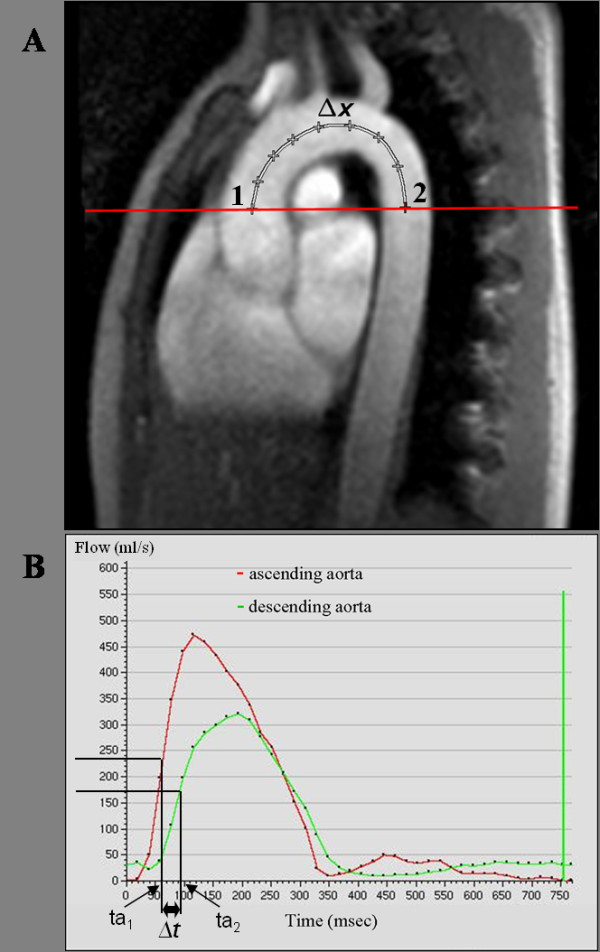
**Aortic PWV, A) Sagittal CMR image that shows the sites where phase contrast cine images were acquired: 1) ascending aorta, 2) descending aorta.** The distance between both locations (Δx) was measured along a midline through the aortic arch. **B**) This graph shows the transit delay (Δt) of the systolic flow curves in the descending relative to the ascending aorta. The transit time (Δt) was determined from the midpoints of the systolic up-slope (ta1 and ta2) on the flow versus time curves. The difference of ta for ascending (ta1) and descending aorta (ta2) locations defined Δt. Pulse wave velocity was estimated as Δx/Δt.

Phase-contrast cine CMR was performed to evaluate aortic PWV and to quantify aortic flow. PWV was assessed in a segment including the ascending aorta, the aortic arch and the proximal descending aorta up to the level of the pulmonary artery bifurcation (Figure 1). The slice plane intersected the ascending aorta at the sinutubular level, and the proximal descending aorta, both at an approximately right angle. Imaging parameters were as follows: FOV 270 × 270 mm, voxel size 1.64 × 1.4 × 7 mm, TR/TE = 4.4/2.7 ms, 40 cardiac phases, velocity encoding = 200 cm/s. To determine the aortic segment length between the two aortic levels, sagittally angulated views of the aortic arch were acquired.

### Image analysis

The images were analyzed with commercially available CMR software (ViewForum release 6.3, Philips Medical Systems, Netherlands).

**Figure 2:**
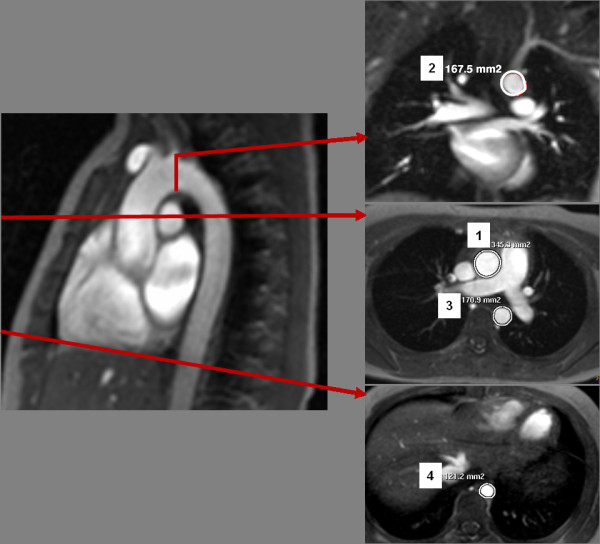
Cross-sectional aortic areas were assessed from axial and coronal CMR images at four different locations of the thoracic aorta: ascending aorta (1), transverse aortic arch (2), aortic isthmus (3), descending aorta above the diaphragm (4).

Aortic cross-sectional areas were determined on axial and coronal gradient-cine images at four positions (Figure 2): ascending aorta, transverse aortic arch, aortic isthmus and descending aorta above the diaphragm. All measurements were made at the time of the maximal distension of the aorta. Aortic cross-sectional areas were preferred compared to diameters, because the aorta is not necessarily circular in all segments.

Aortic distensibility was measured from two-dimensional cine images in the ascending aorta, the transverse aortic arch and at two levels in the descending aorta. The latter were located at the aortic isthmus, and above the diaphragm. Distensibility was calculated [14] as:

$$\begin{array}{c} \text{Distensibility}\;\left(10^{-3}{\text{mm Hg}}^{-1}\right)\\ \;=\left({\text{A}}_{\text{max}}-{\text{A}}_{\text{min}}\right)/{\text{A}}_{\text{min}}\text{x}\;\left({\text{P}}_{\text{max}}-{\text{P}}_{\text{min}}\right),\text{where} \end{array}$$

A_max_ and A_min_ represent the maximal and minimal cross-sectional area of the aorta on cine CMR images, and P_max_ and P_min_ represent the systolic and diastolic blood pressures (in millimetres of mercury), respectively. Blood pressure was obtained non-invasively using a CMR-compatible patient monitor with sphygmomanometer (Invivo Precess™ 3160, Invivo, Orlando, USA). The sphygmomanometer cuff was placed around the right arm.

Aortic flow measurements in the ascending and proximal descending aorta with the CMR phase-contrast technique were used to assess PWV in the aortic arch. Aortic flow versus time curves from phase-contrast cine images were obtained to determine the time delay of the distal flow curve (in the descending aorta), relative to the flow curve in the proximal ascending aorta.

The PWV was calculated by the following equation:

PWV=Δx/Δt,

whereas Δx is the aortic segment length (in meters) along an intra-luminal center-line between the two measurement locations, measured in a sagittally angulated view of the aortic arch, and Δt is the time delay of the distal flow curve, relative to the proximal flow curve (in seconds, Figure 1). Furthermore mean aortic blood flow and peak systolic velocity were assessed in the ascending aorta (Table 2).

**Table 2:** Mean blood flow and maximal flow velocity in the ascending aorta

**Age classes**	**Mean blood flow (l/min)**	**Maximal velocity (cm/s)**
-5 years	2.8 ± 0.1	130.3 ± 2.8
5-10 years	3.9 ± 0.8	131.8 ± 18.3
10-15 years	4.4 ± 0.9	119.7 ± 15.8
15-20 years	6.1 ± 1.4	111.9 ± 23.7
20-25 years	5.6 ± 1.1	121.0 ± 16.0
25-30 years	5.5 ± 1.2	118.5 ± 23.9

Data are expressed as mean and standard deviation.

### Statistical analysis

Statistical analysis was performed using MedCalc® Version 11.5.1.0. The quantitative data were expressed as mean and standard deviation. The Mann–Whitney-*U* test for independent samples was used to compare female and male subgroups. Associations between variables were examined using Spearman’s rank correlation. P values below 0.05 were considered to indicate statistical significance.

Reference curves for the aortic measurements were estimated with the Lambda-Mu-Sigma (LMS)-method from Cole and Green [15,16] for each gender. This method characterizes the age dependent distribution of a target parameter based on a quantile regression fit by three different components: the median (M), the variance (S) and the skewness of the distribution, which is evaluated by an exponential factor (L) from a Box-Cox transformation. L, M and S values can be used to construct reference curves by the following equation:

$${\text{C}}_{\alpha }=\text{M}*{\left(1+\text{L}*\text{S}*{\text{z}}_{\alpha }\right)}^{1/\text{L}}\text{,}$$

where z_α_ is the α-quantile in the standard normal distribution, e. g. z_0,95_ = 1,64. The z-score can be calculated from the LMS values and the measurement value for aortic cross-sectional area, diststensibility or PWV (X):

$$\text{z}-\text{score}=\left[{\left(\text{X}/\text{M}\right)}^{\text{L}}-1\right]/\left(\text{L}*\text{S}\right)\text{.}$$

## Results

The study group characteristics are presented in Table 3. There were no significant differences between female and male subgroups.

**Table 3:** Characteristics of the study population

**Parameter**	**Total study group**	**Female volunteers**	**Male volunteers**	**P value**
	**(n = 71)**	**(n = 41)**	**(n = 30)**	
Age (years)	16.4 ± 7.6	17.5 ± 7.4	14.9 ± 7.7	0.18
Weight (kg)	50.9 ± 21.9	51.4 ±19.3	50.2 ± 25.1	0.73
Height (cm)	156.6 ± 25.6	157.5 ± 23.0	156.1 ± 29.1	0.86
BSA (m^2^)	1.5 ± 0.4	1.5 ± 0.4	1.5 ± 0.5	0.84
BMI (kg/m^2^)	19.4 ± 3.5	19.7 ± 3.4	19.1 ± 3.8	0.57
SBP (mm Hg)	104.9 ± 9.5	103.7 ± 9.3	106.2 ± 9.7	0.48
DBP (mm Hg)	59.2 ± 11.0	58.8 ± 10.9	59.4 ± 11.2	0.91
PP (mm Hg)	45.7 ± 8.4	44.9 ± 6.6	46.8 ± 10.4	0.14

Data are expressed as mean and standard deviation. P-values are from the Mann–Whitney-*U* test.

*BMI*, body mass index; *BSA*, body surface area; *DBP*, diastolic blood pressure; *PP*, pulse pressure; *SBP*, systolic blood pressure.

### Aortic dimensions

**Figure 3:**
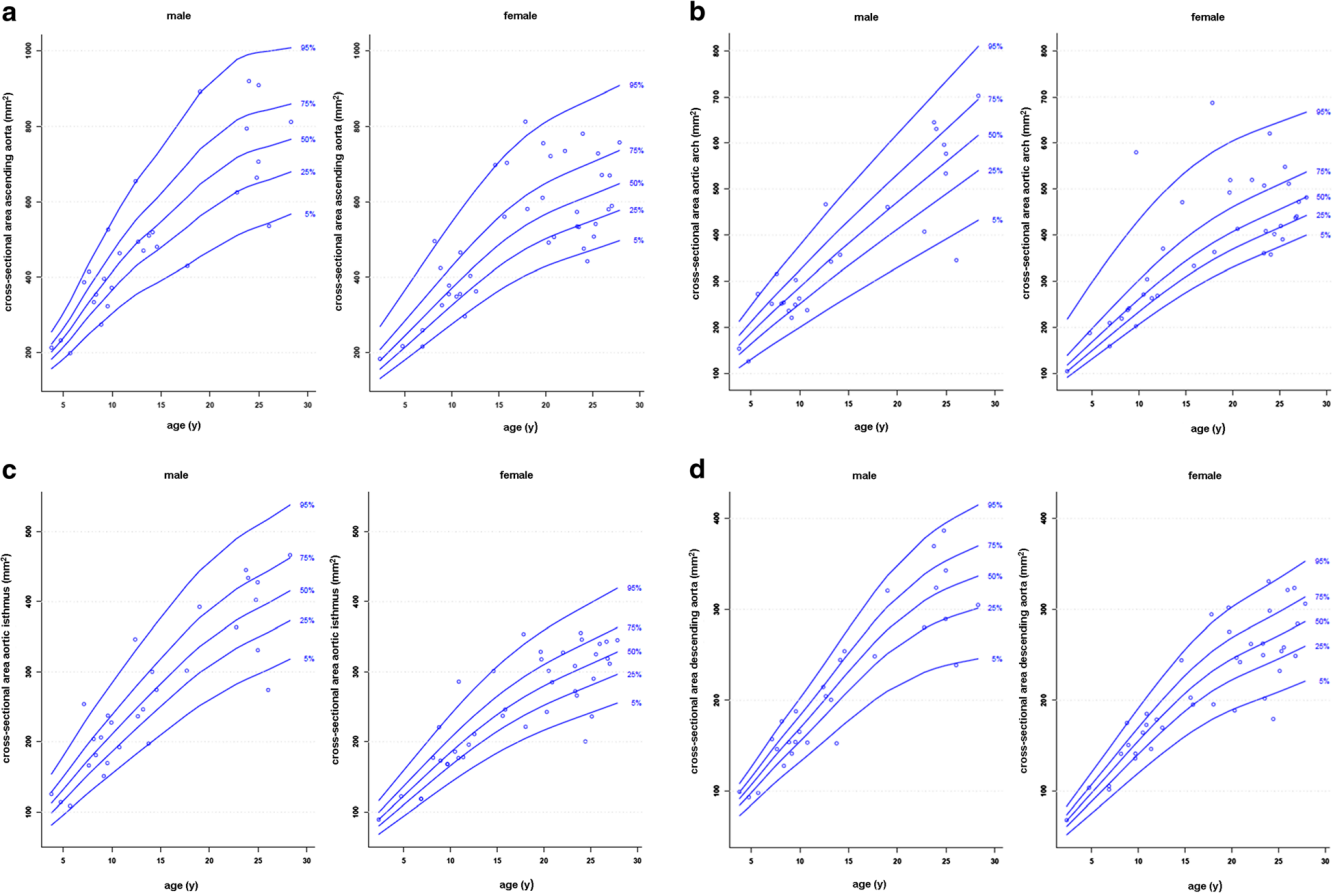
Gender-specific reference percentiles for aortic cross-sectional areas at four locations: a) ascending aorta, b) aortic arch, c) aortic isthmus and d) descending aorta above the diaphragm.

Table 4 shows the mean ± SD of the aortic cross-sectional areas for each position classified by gender. No significant differences were found between males and females. All cross-sectional areas correlated well with the age, height, weight, and body surface area (BSA) (Table 5). Gender specific percentile curves between aortic cross-sectional areas and age are shown in Figure 3. Additionally, Tables 6 and 7 shows the L, M and S values relative to age and gender.

**Table 4:** Cross-sectional areas, distensibility and PWV of the thoracic aorta by gender

**Parameter**	**Total study group**	**Female volunteers**	**Male volunteers**	**P value**
	**(n = 71)**	**(n = 41)**	**(n = 30)**	
Cross-sectional area (mm^2^)				
- Ascending aorta	515.3 ± 186.3	516.1 ± 171.4	514.0 ± 208.3	0.68
- Aortic arch	376.9 ± 148.5	383.0 ± 139.1	368.2 ± 163.7	0.51
- Aortic isthmus	257.9 ± 89.5	250.4 ± 76.2	268.3 ± 105.6	0.64
- Descending aorta above the diaphragm	214.2 ± 75.0	213.6 ± 68.9	214.9 ± 84.0	0.81
Distensibility (10^-3^ mm Hg^-1^)				
- Ascending aorta	8.9 ± 3.6	9.2 ± 3.0	8.5 ± 4.2	0.11
- Aortic arch	7.7 ± 3.3	8.0 ± 3.3	7.2 ± 3.4	0.2
- Aortic isthmus	7.4 ± 2.5	7.7 ± 2.3	7.0 ± 2.7	0.11
- Descending aorta above the diaphragm	8.3 ± 3.0	8.8 ± 3.1	7.7 ± 2.7	0.1
PWV (m/s)	3.6 ± 0.7	3.5 ± 0.6	3.7 ± 0.9	0.14

Data are expressed as mean and standard deviation. P-values are from the Mann–Whitney-*U* test.

*PWV*, pulse wave velocity.

**Table 5:** Correlation of cross-sectional areas with study group characteristics

	**Cross-sectional area (mm**^**2**^**)**			
**Parameter**	**Ascending aorta**	**Aortic arch**	**Aortic isthmus**	**Descending aorta above the diaphragm**
Age (years)	0.80†	0.80†	0.81†	0.87†
Height (cm)	0.84†	0.81†	0.81†	0.82†
Weight (kg)	0.90†	0.85†	0.88†	0.89†
BSA (m^2^)	0.89†	0.84†	0.87†	0.88†

Spearman correlation coefficients rho were calculated for the total study group; †p < 0.01.

*BSA*, body surface area.

**Table 6:** LMS parameters for aortic cross-sectional areas relative to age for girls

	**Ascending aorta**			**Aortic arch**			**Aortic isthmus**			**Descending aorta above the diaphragm**		
**Age**	**L**	**M**	**S**	**L**	**M**	**S**	**L**	**M**	**S**	** L**	** M**	** S**
0	-0,7876	121,1903	0,2152	-2,1750	73,6299	0,2114	0,1033	60,0696	0,1621	0,9371	41,0795	0,1398
1	-0,7876	145,9923	0,2140	-2,1750	92,7307	0,2089	0,1033	72,6142	0,1617	0,9371	52,4930	0,1398
2	-0,7876	170,7944	0,2127	-2,1750	111,8315	0,2064	0,1033	85,1587	0,1613	0,9371	63,9065	0,1398
3	-0,7876	195,5999	0,2114	-2,1750	130,9296	0,2039	0,1033	97,7032	0,1609	0,9371	75,3185	0,1398
4	-0,7876	220,4539	0,2102	-2,1750	149,9904	0,2013	0,1033	110,2465	0,1605	0,9371	86,7100	0,1398
5	-0,7876	245,4281	0,2089	-2,1750	168,9588	0,1988	0,1033	122,7870	0,1601	0,9371	98,0510	0,1398
6	-0,7876	270,5738	0,2076	-2,1750	187,8089	0,1963	0,1033	135,3263	0,1597	0,9371	109,3784	0,1398
7	-0,7876	295,9027	0,2064	-2,1750	206,5696	0,1938	0,1033	147,8724	0,1593	0,9371	120,8531	0,1398
8	-0,7876	321,3290	0,2051	-2,1750	225,2367	0,1913	0,1033	160,3915	0,1588	0,9371	132,5201	0,1398
9	-0,7876	346,5367	0,2038	-2,1750	243,7024	0,1887	0,1033	172,7395	0,1584	0,9371	144,0843	0,1398
10	-0,7876	371,3379	0,2026	-2,1750	261,8643	0,1862	0,1033	184,8049	0,1580	0,9371	155,3776	0,1398
11	-0,7876	395,6874	0,2013	-2,1750	279,6207	0,1837	0,1033	196,5286	0,1576	0,9371	166,4608	0,1398
12	-0,7876	419,5583	0,2000	-2,1750	296,8402	0,1812	0,1033	207,8452	0,1572	0,9371	177,3057	0,1398
13	-0,7876	442,8024	0,1988	-2,1750	313,4236	0,1787	0,1033	218,7232	0,1568	0,9371	187,8984	0,1398
14	-0,7876	465,1326	0,1975	-2,1750	329,2852	0,1761	0,1033	229,1136	0,1564	0,9371	198,1163	0,1398
15	-0,7876	486,2071	0,1962	-2,1750	344,3674	0,1736	0,1033	238,9630	0,1560	0,9371	207,7776	0,1398
16	-0,7876	505,7398	0,1950	-2,1750	358,6387	0,1711	0,1033	248,2461	0,1556	0,9371	216,7982	0,1398
17	-0,7876	523,5836	0,1937	-2,1750	372,0983	0,1686	0,1033	256,9723	0,1552	0,9371	225,1710	0,1398
18	-0,7876	539,7165	0,1924	-2,1750	384,7434	0,1661	0,1033	265,1479	0,1547	0,9371	232,7857	0,1398
19	-0,7876	554,1764	0,1912	-2,1750	396,5833	0,1635	0,1033	272,7929	0,1543	0,9371	239,5830	0,1398
20	-0,7876	567,1207	0,1899	-2,1750	407,6567	0,1610	0,1033	279,9469	0,1539	0,9371	245,6109	0,1398
21	-0,7876	578,7817	0,1886	-2,1750	418,0442	0,1585	0,1033	286,6730	0,1535	0,9371	251,0496	0,1398
22	-0,7876	589,4770	0,1873	-2,1750	427,8971	0,1560	0,1033	293,0630	0,1531	0,9371	256,1671	0,1398
23	-0,7876	599,5300	0,1861	-2,1750	437,3887	0,1534	0,1033	299,2101	0,1527	0,9371	261,1406	0,1398
24	-0,7876	609,3164	0,1848	-2,1750	446,7229	0,1509	0,1033	305,2232	0,1523	0,9371	266,1367	0,1398
25	-0,7876	619,1593	0,1835	-2,1750	456,0570	0,1484	0,1033	311,2003	0,1518	0,9371	271,2515	0,1398
26	-0,7876	629,1747	0,1822	-2,1750	465,4360	0,1459	0,1033	317,2057	0,1514	0,9371	276,5640	0,1398
27	-0,7876	639,3019	0,1810	-2,1750	474,8382	0,1433	0,1033	323,2314	0,1510	0,9371	281,9813	0,1398
28	-0,7876	649,4860	0,1797	-2,1750	484,2530	0,1408	0,1033	329,2650	0,1506	0,9371	287,4341	0,1398
29	-0,7876	659,6776	0,1784	-2,1750	493,6694	0,1383	0,1033	335,2995	0,1502	0,9371	292,8916	0,1398
30	-0,7876	669,8691	0,1772	-2,1750	503,0858	0,1358	0,1033	341,3341	0,1498	0,9371	298,3491	0,1398

*LMS*, L = skewness of the distribution, *M* = median, and *S* = variance.

**Table 7:** LMS parameters for aortic cross-sectional areas relative to age for boys

	**Ascending aorta**			**Aortic arch**			**Aortic isthmus**			**Descending aorta above the diaphragm**		
**Age**	**L**	**M**	**S**	**L**	**M**	**S**	**L**	**M**	**S**	** L**	** M**	** S**
0	0,3091	91,5360	0,1207	0,8668	80,1737	0,1898	0,1267	53,0050	0,1987	1,5823	44,6080	0,1100
1	0,3091	120,6960	0,1274	0,8668	101,7001	0,1897	0,1267	68,7198	0,1974	1,5823	57,0317	0,1115
2	0,3091	149,8560	0,1341	0,8668	123,2265	0,1895	0,1267	84,4347	0,1960	1,5823	69,4554	0,1129
3	0,3091	179,0160	0,1408	0,8668	144,7529	0,1894	0,1267	100,1495	0,1946	1,5823	81,8791	0,1143
4	0,3091	208,1812	0,1475	0,8668	166,2791	0,1893	0,1267	115,8653	0,1932	1,5823	94,3035	0,1158
5	0,3091	238,3791	0,1542	0,8668	187,7555	0,1891	0,1267	131,7743	0,1918	1,5823	106,8833	0,1172
6	0,3091	272,8715	0,1604	0,8668	208,8732	0,1890	0,1267	148,2790	0,1904	1,5823	119,9057	0,1186
7	0,3091	311,2493	0,1660	0,8668	229,2411	0,1888	0,1267	164,9648	0,1891	1,5823	133,0488	0,1201
8	0,3091	346,8686	0,1707	0,8668	248,8676	0,1887	0,1267	180,7624	0,1877	1,5823	145,5984	0,1215
9	0,3091	380,0230	0,1748	0,8668	268,0557	0,1886	0,1267	195,7825	0,1863	1,5823	157,5124	0,1229
10	0,3091	413,8181	0,1782	0,8668	287,2956	0,1884	0,1267	210,6578	0,1849	1,5823	169,3366	0,1244
11	0,3091	446,7220	0,1812	0,8668	306,7317	0,1883	0,1267	225,5414	0,1835	1,5823	181,3951	0,1258
12	0,3091	476,5703	0,1841	0,8668	326,2205	0,1881	0,1267	240,3324	0,1822	1,5823	193,8192	0,1272
13	0,3091	501,7973	0,1870	0,8668	345,4511	0,1880	0,1267	254,6975	0,1808	1,5823	206,4812	0,1287
14	0,3091	524,0769	0,1902	0,8668	364,2701	0,1879	0,1267	268,8289	0,1794	1,5823	219,2939	0,1301
15	0,3091	546,3695	0,1937	0,8668	382,7610	0,1877	0,1267	282,9653	0,1780	1,5823	232,0152	0,1316
16	0,3091	569,8955	0,1972	0,8668	400,9805	0,1876	0,1267	296,9424	0,1766	1,5823	244,3629	0,1330
17	0,3091	594,7536	0,2003	0,8668	418,9724	0,1875	0,1267	310,5833	0,1752	1,5823	256,2294	0,1344
18	0,3091	620,9611	0,2025	0,8668	436,7805	0,1873	0,1267	323,7094	0,1739	1,5823	267,5155	0,1359
19	0,3091	647,1204	0,2034	0,8668	454,4484	0,1872	0,1267	336,0814	0,1725	1,5823	278,0681	0,1373
20	0,3091	670,2706	0,2030	0,8668	472,0177	0,1871	0,1267	347,4348	0,1711	1,5823	287,6962	0,1387
21	0,3091	690,0681	0,2014	0,8668	489,5219	0,1869	0,1267	357,7775	0,1697	1,5823	296,3958	0,1402
22	0,3091	706,8583	0,1990	0,8668	506,9924	0,1868	0,1267	367,1860	0,1683	1,5823	304,2102	0,1416
23	0,3091	720,9831	0,1960	0,8668	524,4603	0,1866	0,1267	375,7366	0,1670	1,5823	311,1823	0,1430
24	0,3091	732,2902	0,1926	0,8668	541,9124	0,1865	0,1267	383,4824	0,1656	1,5823	317,3075	0,1445
25	0,3091	740,4053	0,1889	0,8668	559,3076	0,1864	0,1267	390,6086	0,1642	1,5823	322,5658	0,1459
26	0,3091	747,1815	0,1849	0,8668	576,7470	0,1862	0,1267	397,7409	0,1628	1,5823	327,1568	0,1473
27	0,3091	754,8518	0,1805	0,8668	594,3196	0,1861	0,1267	405,3735	0,1614	1,5823	331,4000	0,1488
28	0,3091	763,4054	0,1758	0,8668	611,9863	0,1860	0,1267	413,3799	0,1601	1,5823	335,4719	0,1502
29	0,3091	772,1960	0,1711	0,8668	629,6783	0,1858	0,1267	421,4867	0,1587	1,5823	339,4979	0,1516
30	0,3091	780,9891	0,1663	0,8668	647,3706	0,1857	0,1267	429,5945	0,1573	1,5823	343,5234	0,1531

*LMS*, L = skewness of the distribution, *M* = median, and *S* = variance.

### Aortic distensibility and PWV

**Figure 4:**
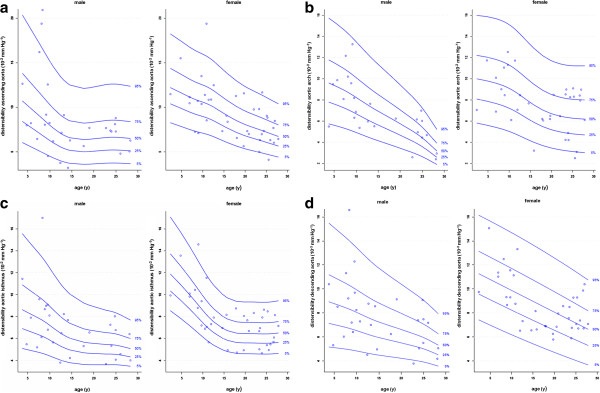
Gender-specific reference percentiles for aortic distensibility at four locations: a) ascending aorta, b) aortic arch, c) aortic isthmus and d) descending aorta above the diaphragm.

**Figure 5:**
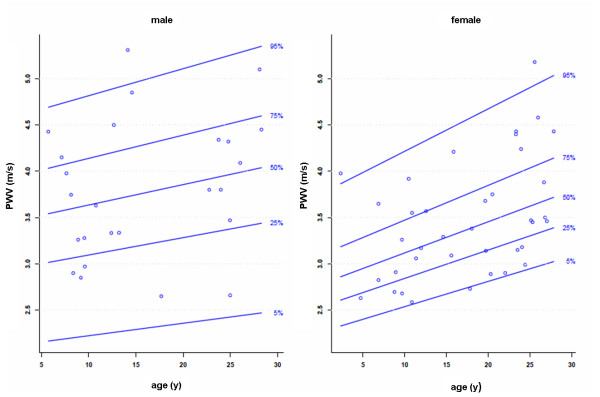
Gender-specific reference percentiles for PWV in the aortic arch.

The mean values of aortic distensibility and PWV are presented in Table 4. Figures 4 and 5 show the gender specific percentiles for aortic distensibility and PWV. In Tables 8, 9, 10 the original L, M and S values for aortic distensibility and PWV are given. An age-related decrease of aortic distensibility was found for all anatomical locations (r = -0.43 to -0.52, p < 0.001). Aortic distensibility also correlated with height (r = -0.47 to -0.62, p < 0.001) and body weight (r = -0.45 to -0.59, p < 0.001), BSA (r = -0.47 to -0.61, p < 0.001). Univariate regression analysis showed a modest association between PWV and the following parameters: age, height, weight and BSA (r = 0.25 to 0.32, p = 0.047 to 0.009).

**Table 8:** LMS parameters for aortic distensibility relative to age for girls

	**Ascending aorta**			**Aortic arch**			**Aortic isthmus**			**Descending aorta above the diaphragm**		
**Age**	**L**	**M**	**S**	**L**	**M**	**S**	**L**	**M**	**S**	** L**	** M**	** S**
0	-0,0721	12,7303	0,2388	0,2825	10,1659	0,2971	-0,2728	12,7827	0,2097	0,3847	11,6928	0,2263
1	-0,0721	12,5028	0,2396	0,2825	10,0908	0,3006	-0,2728	12,3919	0,2100	0,3847	11,5113	0,2297
2	-0,0721	12,2753	0,2403	0,2825	10,0157	0,3041	-0,2728	12,0010	0,2102	0,3847	11,3298	0,2331
3	-0,0721	12,0477	0,2411	0,2825	9,9404	0,3076	-0,2728	11,6098	0,2104	0,3847	11,1483	0,2366
4	-0,0721	11,8176	0,2419	0,2825	9,8619	0,3110	-0,2728	11,2134	0,2106	0,3847	10,9668	0,2400
5	-0,0721	11,5817	0,2427	0,2825	9,7752	0,3145	-0,2728	10,8041	0,2108	0,3847	10,7853	0,2434
6	-0,0721	11,3421	0,2435	0,2825	9,6765	0,3180	-0,2728	10,3772	0,2110	0,3847	10,6038	0,2469
7	-0,0721	11,1121	0,2443	0,2825	9,5629	0,3215	-0,2728	9,9344	0,2112	0,3847	10,4223	0,2503
8	-0,0721	10,9051	0,2451	0,2825	9,4311	0,3250	-0,2728	9,4780	0,2114	0,3847	10,2408	0,2537
9	-0,0721	10,7290	0,2459	0,2825	9,2748	0,3285	-0,2728	9,0114	0,2117	0,3847	10,0593	0,2572
10	-0,0721	10,5679	0,2467	0,2825	9,0871	0,3320	-0,2728	8,5463	0,2119	0,3847	9,8778	0,2606
11	-0,0721	10,3851	0,2474	0,2825	8,8635	0,3354	-0,2728	8,1077	0,2121	0,3847	9,6963	0,2640
12	-0,0721	10,1582	0,2482	0,2825	8,6056	0,3389	-0,2728	7,7159	0,2123	0,3847	9,5149	0,2675
13	-0,0721	9,8884	0,2490	0,2825	8,3257	0,3424	-0,2728	7,3784	0,2125	0,3847	9,3334	0,2709
14	-0,0721	9,5911	0,2498	0,2825	8,0371	0,3459	-0,2728	7,0939	0,2127	0,3847	9,1519	0,2743
15	-0,0721	9,2905	0,2506	0,2825	7,7528	0,3494	-0,2728	6,8625	0,2129	0,3847	8,9705	0,2777
16	-0,0721	9,0033	0,2514	0,2825	7,4850	0,3529	-0,2728	6,6852	0,2131	0,3847	8,7890	0,2812
17	-0,0721	8,7345	0,2522	0,2825	7,2434	0,3563	-0,2728	6,5631	0,2134	0,3847	8,6076	0,2846
18	-0,0721	8,4850	0,2529	0,2825	7,0315	0,3598	-0,2728	6,4885	0,2136	0,3847	8,4262	0,2880
19	-0,0721	8,2574	0,2537	0,2825	6,8508	0,3633	-0,2728	6,4533	0,2138	0,3847	8,2448	0,2915
20	-0,0721	8,0546	0,2545	0,2825	6,6995	0,3668	-0,2728	6,4449	0,2140	0,3847	8,0635	0,2949
21	-0,0721	7,8749	0,2553	0,2825	6,5740	0,3703	-0,2728	6,4447	0,2142	0,3847	7,8822	0,2983
22	-0,0721	7,7106	0,2561	0,2825	6,4710	0,3738	-0,2728	6,4391	0,2144	0,3847	7,7008	0,3017
23	-0,0721	7,5479	0,2569	0,2825	6,3882	0,3772	-0,2728	6,4264	0,2146	0,3847	7,5195	0,3052
24	-0,0721	7,3842	0,2577	0,2825	6,3235	0,3807	-0,2728	6,4171	0,2148	0,3847	7,3382	0,3086
25	-0,0721	7,2113	0,2584	0,2825	6,2744	0,3842	-0,2728	6,4170	0,2150	0,3847	7,1570	0,3120
26	-0,0721	7,0343	0,2592	0,2825	6,2371	0,3877	-0,2728	6,4317	0,2152	0,3847	6,9757	0,3155
27	-0,0721	6,8647	0,2600	0,2825	6,2084	0,3912	-0,2728	6,4666	0,2154	0,3847	6,7944	0,3189
28	-0,0721	6,6951	0,2608	0,2825	6,1806	0,3947	-0,2728	6,5104	0,2156	0,3847	6,6132	0,3223
29	-0,0721	6,5250	0,2616	0,2825	6,1527	0,3981	-0,2728	6,5550	0,2158	0,3847	6,4319	0,3257
30	-0,0721	6,3550	0,2624	0,2825	6,1249	0,4016	-0,2728	6,5997	0,2160	0,3847	6,2506	0,3292

*LMS*, L = skewness of the distribution, *M* = median, and *S* = variance.

**Table 9:** LMS parameters for aortic distensibility relative to age for boys

	**Ascending aorta**			**Aortic arch**			**Aortic isthmus**			**Descending aorta above the diaphragm**		
**Age**	**L**	**M**	**S**	**L**	**M**	**S**	**L**	**M**	**S**	** L**	** M**	** S**
0	-0,1879	12,3602	0,3680	0,0801	10,2881	0,3061	-0,2022	9,4472	0,3533	-0,0079	9,4241	0,3478
1	-0,1879	11,9220	0,3680	0,0801	10,1050	0,3061	-0,2022	9,2308	0,3494	-0,0079	9,2938	0,3441
2	-0,1879	11,4838	0,3680	0,0801	9,9218	0,3061	-0,2022	9,0144	0,3454	-0,0079	9,1635	0,3404
3	-0,1879	11,0456	0,3680	0,0801	9,7386	0,3061	-0,2022	8,7980	0,3415	-0,0079	9,0331	0,3367
4	-0,1879	10,6075	0,3680	0,0801	9,5554	0,3061	-0,2022	8,5816	0,3375	-0,0079	8,9028	0,3330
5	-0,1879	10,1700	0,3680	0,0801	9,3705	0,3061	-0,2022	8,3685	0,3335	-0,0079	8,7730	0,3293
6	-0,1879	9,7343	0,3680	0,0801	9,1780	0,3061	-0,2022	8,1629	0,3296	-0,0079	8,6433	0,3256
7	-0,1879	9,2990	0,3680	0,0801	8,9719	0,3061	-0,2022	7,9576	0,3256	-0,0079	8,5103	0,3219
8	-0,1879	8,8602	0,3680	0,0801	8,7467	0,3061	-0,2022	7,7410	0,3216	-0,0079	8,3694	0,3182
9	-0,1879	8,4151	0,3680	0,0801	8,4980	0,3061	-0,2022	7,4918	0,3177	-0,0079	8,2157	0,3145
10	-0,1879	7,9776	0,3680	0,0801	8,2310	0,3061	-0,2022	7,2033	0,3137	-0,0079	8,0497	0,3108
11	-0,1879	7,5683	0,3680	0,0801	7,9577	0,3061	-0,2022	6,8970	0,3098	-0,0079	7,8764	0,3071
12	-0,1879	7,2051	0,3680	0,0801	7,6865	0,3061	-0,2022	6,6042	0,3058	-0,0079	7,7035	0,3034
13	-0,1879	6,9030	0,3680	0,0801	7,4220	0,3061	-0,2022	6,3480	0,3018	-0,0079	7,5379	0,2997
14	-0,1879	6,6697	0,3680	0,0801	7,1669	0,3061	-0,2022	6,1310	0,2979	-0,0079	7,3831	0,2959
15	-0,1879	6,5089	0,3680	0,0801	6,9220	0,3061	-0,2022	5,9529	0,2939	-0,0079	7,2400	0,2922
16	-0,1879	6,4138	0,3680	0,0801	6,6851	0,3061	-0,2022	5,8175	0,2899	-0,0079	7,1082	0,2885
17	-0,1879	6,3729	0,3680	0,0801	6,4532	0,3061	-0,2022	5,7175	0,2860	-0,0079	6,9844	0,2848
18	-0,1879	6,3745	0,3680	0,0801	6,2234	0,3061	-0,2022	5,6445	0,2820	-0,0079	6,8653	0,2811
19	-0,1879	6,4062	0,3680	0,0801	5,9926	0,3061	-0,2022	5,5886	0,2781	-0,0079	6,7467	0,2774
20	-0,1879	6,4551	0,3680	0,0801	5,7579	0,3061	-0,2022	5,5400	0,2741	-0,0079	6,6246	0,2737
21	-0,1879	6,5111	0,3680	0,0801	5,5167	0,3061	-0,2022	5,4988	0,2701	-0,0079	6,4987	0,2700
22	-0,1879	6,5646	0,3680	0,0801	5,2667	0,3061	-0,2022	5,4678	0,2662	-0,0079	6,3697	0,2663
23	-0,1879	6,6062	0,3680	0,0801	5,0054	0,3061	-0,2022	5,4496	0,2622	-0,0079	6,2384	0,2626
24	-0,1879	6,6277	0,3680	0,0801	4,7261	0,3061	-0,2022	5,4328	0,2583	-0,0079	6,1017	0,2589
25	-0,1879	6,6242	0,3680	0,0801	4,4155	0,3061	-0,2022	5,3874	0,2543	-0,0079	5,9498	0,2552
26	-0,1879	6,5975	0,3680	0,0801	4,0684	0,3061	-0,2022	5,3095	0,2503	-0,0079	5,7759	0,2515
27	-0,1879	6,5577	0,3680	0,0801	3,6929	0,3061	-0,2022	5,2154	0,2464	-0,0079	5,5826	0,2478
28	-0,1879	6,5116	0,3680	0,0801	3,3021	0,3061	-0,2022	5,1114	0,2424	-0,0079	5,3785	0,2441
29	-0,1879	6,4643	0,3680	0,0801	2,9079	0,3061	-0,2022	5,0026	0,2384	-0,0079	5,1722	0,2404
30	-0,1879	6,4170	0,3680	0,0801	2,5137	0,3061	-0,2022	4,8936	0,2345	-0,0079	4,9658	0,2367

*LMS*, L = skewness of the distribution, *M* = median, and *S* = variance.

**Table 10:** LMS parameters for PWV relative to age and gender

	**Male**			**Female**		
**Age**	**L**	**M**	**S**	**L**	**M**	**S**
0	1,4844	3,4147	0,2122	-1,5196	2,7808	0,1468
1	1,4844	3,4367	0,2122	-1,5196	2,8144	0,1469
2	1,4844	3,4587	0,2122	-1,5196	2,8481	0,1469
3	1,4844	3,4808	0,2122	-1,5196	2,8817	0,1469
4	1,4844	3,5028	0,2122	-1,5196	2,9154	0,1470
5	1,4844	3,5248	0,2122	-1,5196	2,9490	0,1470
6	1,4844	3,5469	0,2122	-1,5196	2,9827	0,1470
7	1,4844	3,5689	0,2122	-1,5196	3,0163	0,1470
8	1,4844	3,5909	0,2122	-1,5196	3,0499	0,1471
9	1,4844	3,6129	0,2122	-1,5196	3,0836	0,1471
10	1,4844	3,6350	0,2122	-1,5196	3,1172	0,1471
11	1,4844	3,6570	0,2122	-1,5196	3,1509	0,1471
12	1,4844	3,6790	0,2122	-1,5196	3,1845	0,1472
13	1,4844	3,7011	0,2122	-1,5196	3,2182	0,1472
14	1,4844	3,7231	0,2122	-1,5196	3,2518	0,1472
15	1,4844	3,7451	0,2122	-1,5196	3,2855	0,1473
16	1,4844	3,7672	0,2122	-1,5196	3,3192	0,1473
17	1,4844	3,7892	0,2122	-1,5196	3,3528	0,1473
18	1,4844	3,8112	0,2122	-1,5196	3,3865	0,1473
19	1,4844	3,8333	0,2122	-1,5196	3,4201	0,1474
20	1,4844	3,8553	0,2122	-1,5196	3,4538	0,1474
21	1,4844	3,8773	0,2122	-1,5196	3,4875	0,1474
22	1,4844	3,8994	0,2122	-1,5196	3,5211	0,1475
23	1,4844	3,9214	0,2122	-1,5196	3,5548	0,1475
24	1,4844	3,9434	0,2122	-1,5196	3,5885	0,1475
25	1,4844	3,9655	0,2122	-1,5196	3,6221	0,1476
26	1,4844	3,9875	0,2122	-1,5196	3,6558	0,1476
27	1,4844	4,0096	0,2122	-1,5196	3,6895	0,1476
28	1,4844	4,0316	0,2122	-1,5196	3,7231	0,1476
29	1,4844	4,0536	0,2122	-1,5196	3,7568	0,1477
30	1,4844	4,0757	0,2122	-1,5196	3,7905	0,1477

*LMS*, L = skewness of the distribution, *M* = median, and *S* = variance.

## Discussion

This CMR study describes the quantile distribution of cross-sectional areas, distensibility and PWV of the thoracic aorta in heart-healthy children and young adults between their 3^rd^ and 29^th^ year of life. Defining the normal range for aortic size and bio-elastic properties is an important aid in the early detection of adverse aortic changes.

### Aortic dimensions

Knowledge of the size of the thoracic aorta is important for the treatment of patients with congenital and acquired cardiovascular diseases. CMR allows an exact assessment of the aortic anatomy, independent of acoustic windows, and is therefore an optimal tool to detect anatomic abnormalities of the aorta such as dilatation or aneurysm formation [17]. We provide normal data for aortic cross-sectional areas in the form of percentile curves by age and gender. Normal data for aortic dimensions in children have been reported in various echocardiographic [18], angiocardiographic [19,20] and CMR studies [21,22].

The CMR study from Kaiser et al. [20] reported aortic diameters measured by contrast-enhanced (CE) CMR angiography in 53 children. This method was limited by the fact that CE-CMR images are static and represent a summation of all cardiac phases which may affect a comparison to ECG-gated acquisitions such as in echocardiography or in our study. Furthermore, they measured aortic diameters instead of cross-sectional areas.

Another early CMR-study from Mohiaddin et al. [22] assessed aortic cross-sectional areas from enddiastolic spin echo images in 70 predominantly adult volunteers between the ages of 10 and 83 years. We confirmed their finding that aortic dimensions are positively correlated with age, but our study also covers children younger than 10 years, an age range where aortic dimensions and cardiac structures change rapidly according to somatic growth in prepubertal children [23]. Normal data for aortic cross-sectional areas have been reported by Rammos et al using angiocardiography [19]. As in our study, they showed a good correlation between BSA and aortic cross-sectional areas. However, their reported data are smaller than in our study, which may be mainly caused by the different technique.

However, the data from the mentioned studies are not exactly comparable to our measurements. Most studies used different imaging modalities [18-20]. Furthermore, they report aortic diameters, or measured them in order to calculate cross-sectional areas [18]. CMR allows a direct measurement of aortic cross-sectional areas which is a more accurate approach to assess aortic size, since vessels are not circular in all segments and show an inter-individual anatomic variability.

The observed quantile distributions for aortic dimensions are of clinical value to detect pathologic anatomical changes of the aorta in children and young adults and will serve as reference values for future CMR research studies.

### Aortic distensibility and PWV

This is the first study to provide reference CMR values for aortic distensibility and PWV in children and young adults, in conjunction with measurements of aortic size. The percentile ranges show that aortic distensibility decreases with age, whereas PWV increases with age. Age-associated changes and reference values of aortic distensibility and PWV in children and adults have also been reported in studies using different techniques. Senzaki et al. examined 112 patients with an age range from 6 months to 20 years by cardiac catherization. They showed that the arterial compliance normalized to body surface area significantly decreased with age [24]. The study by Avioli et al. used transcutaneous Doppler techniques to assess aortic PWV in subjects with an age range from 3 to 89 years. In their study aortic PWV significantly increased with age [25]. Another study measured PWV with ultrasound methods in 206 patients aged 0–15 years. Their median PWV was 3.04 m/s which is comparable to our data. However, in contrast to our study PWV was independent of age, which may be caused by the young age of their study group [26]. CMR assessment of aortic PWV showed good agreement with PWV obtained from invasive pressure measurements as the gold standard [27]. Unlike in ultrasound CMR is not limited to acoustic windows and does not only provide an estimation of aortic PWV [1,27].

The aorta acts as a conduit delivering blood to the peripheral organs and transforms the pulsatile effect caused by ventricular ejection into a continuous blood flow [1]. As shown aortic distensibility decreases and PWV increases during age. The decreasing aortic elasticity observed in our young cohort may be related to normal structural wall changes during aging [28]. An increase in intimal-medial thickness after birth has been demonstrated in an earlier study [28]. The aortic elastic properties depend largely on the presence of elastic fibres in the vessel wall, which have a maximum rate in the perinatal period followed by a fast decrease already during childhood [29]. Besides these developmental changes aortic wall mechanics and stress seems to play an important role in aortic stiffening. In the course of a lifetime the human aorta will undergo billions of cycles of expansion and contractions. This cyclic mechanical stress leads to fragmentation of elastic fibres and causes a transfer of stress to the stiffer collagen fibres. The loss of elastin results in a reduction of aortic elasticity [30]. In adults, decreased aortic elasticity has adverse effects on cardiac systolic and diastolic function, due to increased left ventricular afterload and myocardial oxygen consumption as well as impaired coronary perfusion [31].

 Our data may be of interest in various diseases and pathological conditions that can affect aortic bioelasticity already in children and young adults. Impaired aortic bioelasticity has been reported for instance in patients with Marfan syndrome [14], tetralogy of Fallot [5], Turner’s syndrome [32] and aortic coarctation [4]. In a recently published study we could show that patients with hypoplastic left heart syndrome have severly reduced aortic distensibility [3]. Furthermore, some functional vascular parameters are impaired in obese children [33]. Considering the increasing use of CMR for non-invasive scientific and clinical studies, the presented data may help in evaluating aortic bioelastic function and cardiovascular risk stratification with these diseases.

### Limitations

As it is difficult to recruit healthy children as volunteers for a CMR study, the sample size of our cohort is small in comparison to echocardiographic studies but fulfills the demand for statistical evaluation.

## Conclusions

We provide percentiles for aortic cross-sectional areas, aortic distensibilty and PWV for children at various ages. They can be of clinical value in patients with various cardiac and vascular diseases and may serve as reference values for further CMR research studies.

## Competing interests

The authors declare that they have no competing interests.

## Authors’ contributions

IV and CR developed the concept and design of the study, collected the data and analyzed the results. They also drafted the manuscript and had the primary responsibility for the final content. JH from the Department for Medical Informatics and Statistics participated substantially in statistical analysis and data interpretation. EP and CH assisted substantially in data acquisition and analysis. DDG, JHH and CP revised the article for important intellectual content. HHK and MJH participated mainly in the design of the study and the critical revision of the manuscript. All authors have read and approved the final manuscript.
